# The relationship of life-course patterns of adiposity with type 2 diabetes, depression, and their comorbidity in the Northern Finland Birth Cohort 1966

**DOI:** 10.1038/s41366-022-01134-y

**Published:** 2022-05-13

**Authors:** Priyanka Choudhary, Justiina Ronkainen, Rozenn Nedelec, Mimmi Tolvanen, Estelle Lowry, Jouko Miettunen, Marjo-Riitta Jarvelin, Sylvain Sebert

**Affiliations:** 1 Center for Life Course Health Research, Faculty of Medicine, University of Oulu, Oulu, Finland; 2 Queen’s University Belfast, Belfast, UK; 3 Medical Research Center Oulu, Oulu University Hospital and University of Oulu, Oulu, Finland; 4 Department of Epidemiology and Biostatistics, School of Public Health, Imperial College London, London, UK; 5 MRC-PHE Centre for Environment and Health, School of Public Health, Imperial College, London, UK; 6 Department of Life Sciences, College of Health and Life Sciences, Brunel University London, Kingston Lane, Uxbridge, Middlesex UK

**Keywords:** Type 2 diabetes, Obesity, Risk factors

## Abstract

**Objectives:**

Type 2 diabetes (T2D) and comorbid depression challenges clinical management particularly in individuals with overweight. We aim to explore the shared etiology, via lifecourse adiposity, between T2D and depression.

**Methods:**

We used data from birth until 46years from Northern Finland Birth Cohort 1966 (*n* = 6,372; 53.8% females). We conducted multivariate analyses on three outcomes: T2D (4.2%), depression (19.2%) and as comorbidity (1.8%). We conducted (i) Path analysis to clarify time-dependent body mass index (BMI) related pathways, including BMI polygenic risk scores (PRS); and (ii) Cox regression models to assess whether reduction of overweight between 7years and 31years influence T2D, depression and/or comorbidity. The models were tested for covariation with sex, education, smoking, physical activity, and diet score.

**Results:**

The odd ratios (OR) of T2D in individuals with depression was 1.68 [95% confidence interval (CI): 1.34–2.11], and no change in estimate was observed when adjusted for covariates. T2D and comorbidity showed similar patterns of relationships in the path analyses (*P* < 0.001). The genetic risk for obesity (PRS BMI) did not show direct effect on T2D or comorbidity in adulthood but indirectly through measures of adiposity in early childhood and mid-adulthood in the path analysis (*P* < 0.001). Having early-onset of overweight at 7years and 31years showed highest risk of T2D (OR 3.8, 95%CI 2.4–6.1) and comorbidity (OR 5.0, 95%CI 2.7–9.5), with mild-to-moderate attenuation with adjustments. Depression showed no significant associations.

**Conclusions:**

We found evidence for overweight since childhood as a risk factor for T2D and co-morbidity between T2D and depression, influenced moderately by lifestyle factors in later life. However, no shared early life adiposity related risk factors were observed between T2D and depression when assessed independently in this Finnish setting.

## Introduction

Type 2 diabetes (T2D), depression and obesity are among the leading cause of morbidity and mortality globally [1]. The vulnerability of the populations with these conditions has grabbed significant attention in the COVID-19 pandemic as they have required intensive clinical care and had the worse mortality outcomes [2, 3]. The prevalence of diabetes has quadrupled since 1980 to reach 10.5% in 2021 [4] and depression alone accounts for 12% of total years lived with disability, affecting approximately 300 million people annually [5]. A steep rise was seen in obesity levels since 1980’s and worryingly, over 23% of children worldwide are in stages of obesity or overweight [6].

Multimorbidity (co-occurrence of two or more conditions) is observed as a common phenomenon in the ageing population; often challenging clinical management of the diseases. Epidemiological studies indicate that individuals with depression have up to a 60% higher risk of T2D and up to 23% of individuals with T2D meet the criteria for depression [7, 8]. This co-occurrence is expected not to be at random. They are often observed as part of a bi-directional relationship, attributable to common lifestyle predictors such as physical activity (PA), smoking, and diet. These shared environmental pathways may act as a hub, linking the comorbidity [9]. Nevertheless, the proportion of shared etiology between T2D and depression and their early origins remain unknown. The early life origin of T2D due to adverse in-utero conditions and lower birthweight is well-established [10], but whether fetal life plays a part in determining biological susceptibility to depression is less clear [11].

The co-occurrence of T2D and depression is exacerbated in individuals with obesity [12]. This raises the question of how much the relationship between T2D and depression is explained by time-dependent changes in adiposity and other lifestyle predictors such as education, smoking, diet, and PA. Two studies found an association between adolescent obesity and depression [13, 14], but other studies found inconsistent associations at different childhood ages [15, 16]. Similarly, childhood overweight was associated with increased risk of T2D when continued into adulthood but they found no role of education or smoking in this association [17]. Otherwise, genetically both T2D and depression are heritable and postulated to share 25% genetic liability [18].

Previous studies have mainly used retrospective [19] or prospective [20] analysis, but no studies have explored the relationship of T2D and depression as a comorbidity in a lifecourse approach. Therefore, we aimed to understand the temporal relationship of longitudinal adiposity measures connecting T2D and depression using two different methods. Our objectives were: 1) to model the plausible direct and indirect pathways using clinical measures and polygenic risk score (PRS) for BMI on the three conditions, and 2) to assess whether remission of overweight from childhood to adulthood lowers the risk of T2D, depression or both using survival analysis.

## Methods

We used data from the Northern Finland Birth Cohort 1966 (NFBC1966) as described previously [21]. The design of the data collections used in the analyses are described in the study flowchart (Supplementary Fig. 1). The population studied was a non-selected sample of 12137 live-born children, covering 96.3% of all the births in the year 1966 within the catchment area (Provinces of Oulu and Lapland). The participants have been followed up since then; prospectively, with postal questionnaires and clinical visits at the age of 31 years and 46 years and additional data is available from linkage to health and social registers. We included the information on exposures (birthweight, BMI and overweight patterns) and covariates using follow-up data during pregnancy, 7 and 31 years. The information on outcomes (T2D, depression and comorbidity) was obtained from ages 32–50 years using follow-up data at 46 years and data collected continuously through national registers between 32–50 years. We excluded individuals born preterm (gestational age (GA) < 37 weeks; *n* = 1127), multiple births (*n* = 318), individuals with self-reported depression (*n* = 398) and with diabetes at 31 years follow-up (*n* = 15) to exclude juvenile detection of diabetes.

All participants included in this study provided written informed consent for the use of both prospectively collected data and its linkage to national health and social registers data. The studies received ethical approval from Northern Ostrobothnia Hospital District Ethical Committee 94/2011 (12.12.2011).

### Exposures: lifecourse estimators of adiposity from birth onwards

The measures of adiposity considered in this study included birthweight standard deviation scores (SDS) by GA and sex [22], BMI at 7years and 31years. Birthweight was measured in grams by a nurse immediately after birth. GA was derived from the mother’s last menstrual period. BMI (kg/m^2^) was measured from participants’ height (cm) and weight (kg) collected from primary healthcare, school health records at 7 years (min 7.00 years and max 7.99 years) and on-site clinical examinations at 31 years and 46 years. Additionally, DNA was extracted from participants at 31 years and genotyped to calculate a PRS BMI (*n* = 3320), using weighted sum of BMI increasing alleles, as described previously [23].

### Overweight patterns

Overweight was defined in accordance with the World Health Organization [24] and sex-specific criteria at 7 years and adulthood were: BMI ≥ 17.38 kg/m^2^ at 7 years and ≥25 kg/m^2^ in adulthood [25]. Combinations of the overweight status at 7 years and 31 years were used to define individual patterns: ‘no overweight (*n* = 4476)’, ‘remission’(overweight at 7 years but not at 31 years *n* = 252)’, ‘late onset’ (overweight first measured at 31 years *n* = 1423)’ and ‘early onset’ (continued overweight since age 7 years *n* = 221).

### Outcomes

Multivariate analysis was conducted using three main outcomes: T2D, depression, and both as comorbidity. Participants with T2D (*n* = 268) were defined based on:

- Self-reported diagnosis at 46 years
- In and out-patient hospital registers (ICD 9 E11)
- Medication registers (ATC code A10BA02) from Social Insurance Institution of Finland including in and outpatient information collected from 1998 to 2016 (between ages 32 years to 50 years)
- Participants clinical examination including measures of fasting plasma glucose greater than 7 mmol/L or 2 hr postload glucose (PG) using oral glucose tolerance test (OGTT) > 11.1 (mmol/L) [26].

For the OGTT, blood samples were taken after an overnight fast from the participants at 46 years. Analyses of plasma glucose were conducted within 24 hr with a glucose dehydrogenase method (Granutest 250, Diagnostica Merck) and serum insulin assay samples were stored at −20 °C and analyzed within 7 days of sampling. Participants without previous diabetes diagnosis underwent OGTT. Blood samples were taken 0, 0.5, 1, and 2 hr after glucose intake and colorimetric assay was used to determine concentration of plasma glucose (mmol/L). The period for diagnosis of T2D started on 1st January 1996, or at the age of 30 years, whichever was later, and ended on the date of diagnosis, death, emigration, loss of follow-up, or December 31, 2016, i.e., by the age of 50 years, whichever happened first. Participants who had depression were excluded from this group.

Similarly, information on depression (*n* = 1224) was collated from three data sources:

- Self-reported diagnosis at 46 years
- Clinical diagnosis from hospital registers from 1998 to 2016 (between ages 32 years to 50 years)
- Self-reported information from postal questionnaire using Beck Depression Inventory items at 46 years. The total raw scores were calculated by adding up all items (range 0–60) and the recommended clinical cutoff score >14 was used.

Participants with T2D were excluded from this group. We did not consider anti-depressant medications in this group as anti-depressants may be used for conditions other than depression.

Comorbidity was defined as participants having both T2D and depression (*n* = 116).

### Statistical analysis

#### Covariates

Smoking, education status, PA and diet are important socio-economic and lifestyle-related predictors of T2D and depression and were included as covariates. All were self-reported through postal questionnaire at 31 years follow-up. Smoking status was categorized as ‘non-smokers’, ‘former/occasional smokers’ and ‘smokers’; and education as ‘basic-elementary’, ‘secondary’ and ‘higher’. PA was derived from questions on frequency and duration of light and brisk PA. The weekly averages of metabolic equivalent of a task (MET) minutes were calculated by multiplying PA volume (duration*frequency) by its intensity (3*light PA METs and 5*brisk PA METs) [27]. Diet score was based on methods commonly used in Finnish surveys of health behaviors. As described previously diet was considered unhealthy when it included daily consumption of high saturated fats, and less frequent (≤2/week) consumption of fiber rich food [28].

We conducted logistic regression to assess association between T2D and depression using SAS (Version 9.4, SAS Institute Inc., Cary, NC, USA). The crude model was sequentially adjusted for education, smoking, PA, unhealthy diet score and sex. Multivariate path analysis was conducted using AMOS ver. 7 to estimate the total, direct and indirect effect of each path variable and model evaluated using model fit indices. The path variables were birthweight, BMI measures and PRS BMI for underlying genetic influence on the three multivariate outcomes. To account for missing data, modelling was based on maximum likelihood estimation, which determines parameters of the distribution under the assumptions that observed data are the most probable and that missing data are at random, giving more robust estimates. A theoretical model of all potential pathways is presented in the Supplementary Fig. S2.

Finally, the relationship between the patterns of overweight and three outcomes were estimated using competing-risks model based on Cox proportional hazards regression. Covariates for adjusted model included sex, smoking, education, low PA, and unhealthy diet score. The proportional hazard assumption of the Cox model was met based on scaled Schoenfeld residuals and plots. The test for each of the covariate and the global test was statistically non-significant which showed assumption of the proportional hazard to be supported (Supplementary Table S1). The models were further stratified by sex (Supplementary Table S2).

#### Additional analysis

Categorized birthweight and underweight at 7 years and 31 years were assessed with three outcomes to elucidate the non-linear relationships (Supplementary Tables S3 and S4). The difference in PRS BMI between the three categories of overweight was also assessed (Supplementary Table S5).

## Results

Among 6372 study participants was a prevalence of 4.2% of T2D, 19.2% of depression and 1.8% with their comorbidity (Table 1). We observed a higher prevalence of T2D in males (58.2%) and of depression in females (65%). Participants with comorbidity had significantly lower birthweight, highest percentage of smoking status, highest unhealthy diet score and lowest PA level compared to other groups (*P* ≤ 0.02). Among the adiposity measures, participants with T2D and comorbidity had significantly higher BMI at 7 years, 31 years and 46 years, overweight at 31 years only and both ages (7 and 31 years) (*P* ≤ 0.0001).

**Table 1:** Descriptive characteristics of the study population.

	Reference population (*n* = 4764)		Type 2 diabetes only (*n* = 268)		Depression only (*n* = 1224)		Comorbidity (*n* = 116)		*P*-value
Offspring	Observations (*N*)	Mean (SD)/ median (IQR) or *N* (%)	Observations (*N*)	Mean (SD)/ median (IQR) or *N* (%)	Observations (*N*)	Mean (SD)/ median (IQR) or *N* (%)	Observations (*N*)	Mean (SD)/ median (IQR) or *N* (%)	
Sex									
Females	4764	2460 (51.6%)	268	112 (41.8%)	1224	794 (64.9%)	116	60 (51.7%)	<0.0001
Males	4764	2304 (48.4%)	268	156 (58.2%)	1224	631 (35.1%)	116	56 (48.3%)	<0.0001
Birth weight (g)	4764	3555 (503)	268	3497 (464)	1224	3537 (491)	116	3447 (545)	0.02
Gestational age (weeks)	4764	40.3 (1.5)	268	40.3 (1.5)	1224	40.4 (1.4)	116	40.3 (1.5)	0.65
BMI 7 years (kg/m^2^)	2875	15.6 (1.8)	155	16.1 (2.2)	721	15.6 (1.8)	67	16.5 (2.4)	<0.0001
BMI 31 years (kg/m^2^)^a^	3162	24.3 (3.9)	182	29.1 (5.9)	780	24.5 (4.2)	78	29.0 (5.8)	<0.0001
BMI 46 years (kg/m^2^)	3885	26.4 (4.4)	214	32.8 (5.9)	1038	27.2 (5.1)	90	33.2 (7.5)	<0.0001
Overweight at 7 years	2875	179 (6.2%)	268	14 (5.2%)	1224	53 (4.3%)	116	6 (5.2%)	<0.0001
Overweight at 31 years	4764	998 (21%)	268	112 (41.8%)	1224	262 (21.4%)	116	51 (44%)	<0.0001
Overweight at both 7 years and 31 years	4764	148 (3.1%)	268	21 (7.8%)	1224	42 (3.4%)	116	10 (8.6%)	<0.0001
Fasting glucose at 31 years (mmol/L)	3124	5 (0.6)	182	5.5 (1.7)	766	4.9 (0.5)	77	5.7 (1.9)	<0.0001
Fasting insulin at 31 years (uIU/mL)^a^	3106	8.2 (4.5)	181	13.9 (14.5)	764	8.3 (7.9)	78	10.9 (5.7)	<0.0001
2 hr PG measured at 46 years^a^	3375	5.7 (1.4)	111	9.5 (3)	877	5.8 (1.5)	43	10.1 (3.7)	<0.0001
Smokers at 31 years	4247	1080 (25.4%)	235	88 (37.5%)	1065	320 (30.1%)	95	44 (46.3%)	<0.0001
Education at 31 years	3762	-	202	-	896	-	86	-	0.02
Basic elementary	-	156 (4.1%)	-	14 (6.9%)	-	56 (6.3%)	-	5 (5.8%)	-
Secondary	-	2933 (77.9%)	-	165 (81.7%)	-	719 (80.3%)	-	72 (83.2%)	-
Higher	-	673 (17.9%)	-	23 (11.4%)	-	121 (13.5%)	-	9 (10.5%)	-
Physical activity (MET hr/wk)^a^	4170	13.8 (0.8)	229	11.3 (0.8)	1051	12.3 (1.3)	93	9.8 (0.8)	<0.0001
Unhealthy diet score	4205	2.03 (0.31)	227	2.22 (0.59)	1054	2.12 (0.30)	92	2.31 (0.66)	0.002
C-Reactive Protein^a^	3539	1.4 (0.63)	211	3.4 (0.29)	945	2.0 (0.56)	86	3.9 (0.29)	<0.0001

*P*-value for differences between groups were calculated using *t-*test and one-way non-parametric test.

*BMI* body mass index, *PG* postload glucose, *MET* metabolic equivalent of a task

^a^Values are percentages for categorical variables, mean (SD) for continuous variables with normal distribution and median (IQR) for skewed variables.

The odds ratio (OR) for T2D in individuals with depression was 1.68 [95% CI: 1.34,2.11], and no change in estimate was observed when adjusted for covariates (Table 2).

**Table 2:** Association between type 2 diabetes and depression and stepwise adjustments for the covariates measured at 31 years.

	Type 2 diabetes	
	OR (95% CI)	*P*-value
Depression	1.68 (1.34, 2.11)	<0.0001
+ Sex	1.79 (1.42, 2.26)	<0.0001
+ Adult education	1.75 (1.34, 2.28)	<0.0001
+ Adult smoking	1.56 (1.22, 2.00)	<0.0001
+ Physical activity	1.57 (1.22, 2.01)	0.0005
+ Diet score	1.60 (1.24, 2.05)	0.0003
Fully adjusted	1.69 (1.28, 2.23)	0.0002

Fully adjusted includes sex, adult education, adult smoking, low physical activity, and unhealthy diet score at 31 years. *P-value < 0.05. In the calculation of odds ratio (OR) participants who never had type 2 diabetes, were used as reference group. Estimates to be interpreted as the odds of having the outcome with one unit change in the exposure.

*CI* Confidence Interval, *OR* Odds Ratio

### Path analysis

**Fig. 1:**
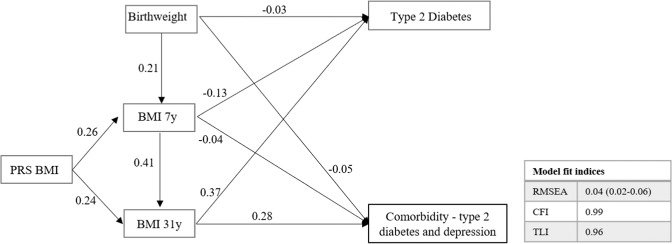
Path diagram illustrating the relationship between adiposity measures over the lifecourse with two outcomes. The values in the path diagram are regression coefficients showing a significant direct effect (*P* ≤ 0.0001) between the variables. Estimates should be interpreted as one standard deviation increase in birthweight is associated with an increase of 0.21 units in BMI at 7 years. No statistically significant pathways were observed with depression. The estimates for the total, direct and indirect standardized effects of the pathways are presented in Supplementary Table S6. BMI body mass index, CFI comparative fit index, PRS polygenic risk score, RMSEA root square error of approximation, TLI Tucker–Lewis index.

Similar patterns of pathways were noticed between T2D and comorbidity (Fig. 1 and Supplementary Table S6). There were no statistically significant pathways with depression. Birthweight showed a negative direct effect on T2D and comorbidity, while a mild positive indirect effect was observed to be mediated by BMI at 7 years and 31 years, however, the total effect remained negative (ß:−0.02; *P* ≤ 0.0001). Similarly, 7 years BMI showed a negative direct effect on T2D and comorbidity while the total effect mediated by BMI at 31 years was positive. BMI at 31 years showed the highest direct effect on both T2D (ß: 0.37; *P* ≤ 0.0001) and comorbidity (ß: 0.28; *P* ≤ 0.0001). PRS BMI did not show any direct effect but did show a positive indirect effect mediated through BMI 31 years on T2D (ß: 0.08; *P* ≤ 0.0001) and comorbidity (ß: 0.03; *P* ≤ 0.0001).

### Cox proportional hazard analysis

**Fig. 2:**
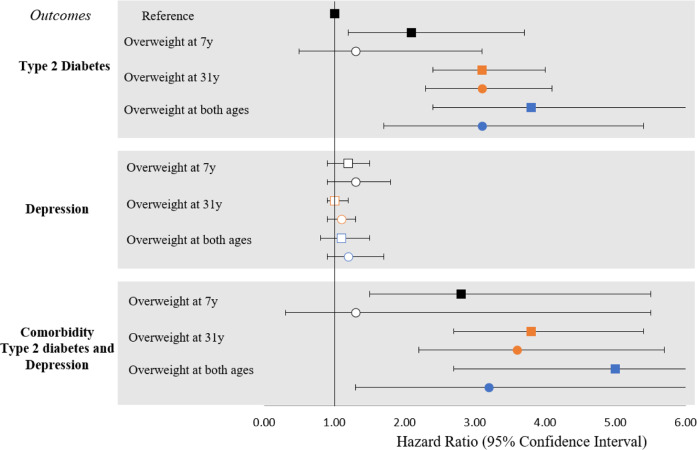
Hazard risk ratio of patterns of overweight over the lifecourse with the three outcomes: type 2 diabetes, depression, and comorbidity. Boxes represent model 1: unadjusted; and circles represent model 2: adjusted for sex, low education, smoking, low physical activity, and unhealthy diet score at 31 years. In the calculation of hazard ratio, participants who had never been overweight were used as reference group. White box and circle denote *P* ≥ 0.05 and coloured box and circle denotes *P* ≤ 0.05. Estimates to be interpreted as the risk of outcome with unit change in the exposure.

The risk of T2D and comorbidity was similar, and in a positive direction with the overweight patterns (Fig. 2, Supplementary Tables S2 and S7). However, the risk of depression was the same for participants who had not been overweight at any time, showing no significant associations. Compared to participants with no history of being overweight, the highest risk of T2D was when participants had persistent early onset of overweight at both 7 years and 31 years (HR: 3.8, 95%CI: 2.4–6.1), followed by overweight at 31 years only (HR: 3.1, 95%CI: 2.4–4.0) and overweight at 7 years only (HR: 2.1, 95%CI: 1.2–3.7). However, upon adjustments for education, smoking, PA, and diet score, the risk of T2D diminished to no significance with overweight at 7 years and showed mild attenuation in the case of overweight at both 7 years and 31 years (HR: 3.1; 95%CI: 1.7, 5.4).

Similarly, the highest risk of comorbidity was among participants who were consistently overweight at both 7 years and 31 years (HR: 5.0; 95%CI: 2.7, 9.5), followed by overweight at 31 years only (HR: 3.8; 95%CI: 2.7, 5.4), and overweight at 7 years only (HR: 2.8; 95%CI: 1.5, 5.5). Upon adjustments, the risk to comorbidity diminished with overweight at 7 years and moderately attenuated with overweight at both 7 years and 31 years (HR: 3.2; 95%CI: 1.3, 7.6) but no change was observed with overweight at 31 years. The stratified analysis showed that females with overweight patterns had up to two-fold higher risk of T2D and comorbidity than males (HR 3.2–5.8 for females and 2.1–4.1 for males) (Supplementary Table S2).

## Discussion

To the best of our knowledge, this is the first study to provide longitudinal insight on the distinctive pathways and temporal relationship of adiposity measures with T2D and depression independently, and as a comorbidity. In our Finnish cohort we found that T2D, and depression do not share early life adiposity related risk factors when present independently. Nevertheless, the risk was increased when both co-occurred as a comorbidity which is likely explained by common lifestyle factors.

Our study supports evidence for an association between T2D and depression. Participants with depression had two times higher odds to also have T2D in comparison to those without depression, consistent with previous evidence [7]. One of the more common hypothesis to date posits that depression and T2D are more likely to be coincidental because of shared environmental and lifestyle risk factors (e.g., smoking, alcohol, physical inactivity, high caloric intake, appetite loss and low socioeconomic status). It is suggested that being a probable cause of each other they may perpetuate a vicious cycle where both conditions worsen each other [29]. However, there is also evidence that they could share several biological risk factors. Insulin-like growth factors play a critical role in determining body growth and are also located in areas of the brain responsible for cognition. Also, hypo and hyper-glycemia affect brain function in areas of cognition and mood. Both conditions associate with hypothalamic-pituitary-adrenal dysfunction which manifests as impaired glucocorticoids sensitivity and enhanced inflammation [30].

In our study the prevalence of T2D was 4.2% which is less than the estimated age-standardized prevalence of T2D in Finland at 8% and 10% in females and males [31]. This difference may be attributed to attrition in participation rate at 46 years follow-up as reported in the cohort profile [21]. On the other hand, the prevalence of depression was 19.2% in our study. In the Finnish Health 2011 survey the estimated prevalence in the general population was 9.6% and 12% for the age group 45–54 years [32]. This is comparable, as in our study we included diagnoses from an 18-years period (ages 32 to 50 years) as well as taking a diverse approach using different measures of depression and depressive symptoms in order to capture the heterogeneity of the condition, while in the general population depression is evaluated using a clinical diagnosis.

### Type 2 diabetes

We observed an inverse association between birthweight and T2D, in line with previous evidence [10]. Our finding in a contemporary population further supports a causal association between lower birthweight and T2D. NFBC1966 is a unique cohort in which participants were born two decades after the end of World War II and reached adulthood just before the global nutrition transition and the start of the obesity epidemic in the 1980–1990s. Historically, in the epidemiology of T2D, World War II created an exogenous effect among children exposed to wartime famines or adverse social conditions. This is exemplified by the Dutch Famine Birth Cohort Study [33], Chinese famine [34] and systematic review [10], highlighting that lower birthweight due to sub-optimal in-utero exposures was associated with development of T2D in later life.

The results from our study are in line with previous studies showing linear trends in association between BMI trajectories since childhood and T2D, with strongest effect in adulthood [35]. In our path analysis BMI at 31 years mediated the indirect influences of birthweight and BMI at 7 years on T2D. Importantly, the total negative effect of birthweight infers that birthweight is a strong early life predictor, irrespective of postnatal determinants in modulating the risk of T2D. The increased strength of association with age may also reflect the latent association of underlying intrauterine programming which is plausible to become more apparent with age. Additionally, PRS BMI showed a strong direct effect on BMI at both 7years and 31 years, and a small indirect effect on T2D when mediated by BMI measures. Alves et al. noted shared genetic determinants of child and adult BMI but not infant BMI and these traits did not correlate with T2D [23]. Authors argued that the role of genotype and environment in BMI heritability increases with age. Many genetic variants associated with obesity appear to be involved in pathways affecting energy homeostasis; however, the identified variants have explained only 15–20% of the heritable variance of T2D [36].

In terms of overweight patterns over the lifecourse, individuals with early onset of overweight which continued into adulthood held the highest risk for T2D and comorbidity. On similar lines, a Danish study on 62,565 men showed an increased risk of T2D when overweight continued until puberty or later ages [17]. Another British birth cohort study of 45 years follow-up identified earlier onset of overweight to be associated with impaired glucose metabolism [37]. However, the Danish study only included men with the latest BMI available at 26 years and in the British birth cohort T2D diagnosis was missing. Upon stratification in our study, we observed that females with overweight had up to two-fold higher risk of T2D and comorbidity than males (Supplementary Table S2). Our study took the novel approach of including the most common determinants of T2D and depression, i.e., education, smoking, low PA, and unhealthy diet which is lacking in previous studies. These lifestyle factors diminished association with overweight at 7years and highly attenuated the association of overweight at 7 years and 31 years hence suggesting that the risk can be reversed at an early as well as later age through lifestyle exposures/factors. This also shows that social and lifestyle factors outweigh the influence of adult adiposity and underlie the pathways leading to T2D and comorbidity.

### Depression

In contrast to T2D, participants with depression alone did not associate with any of the early life adiposity-related measures. To explore further, we tested the association of underweight patterns at 7 years and 31 years with depression. The associations attenuated when adjusted for education, smoking, PA, and diet (Supplementary Table S4). Also, this may rather be a reverse association and consequence of depression on BMI due to loss of appetite and general interest in wellbeing. These findings indicate that T2D and depression in our population may have independent pathways of origin and show absence of a shared link via adiposity channel. Moreover, genetic studies found no overlap between obesity and depression variants [38]. Nevertheless, it is important to keep in mind that these findings could be specific to this population, as dynamics of health may vary largely between different settings and populations. Our findings require further replication in diverse populations with longitudinal data.

### Comorbidity

We observed a similar relationship and pathways for T2D-depression comorbidity with adiposity measures as was seen with T2D alone, but at greater magnitude. In our study, we cannot fully differentiate between depression and T2D-related distress. However, previous studies have reported that most individuals with depressive symptoms are not necessarily clinically depressed but rather have increased distress associated with the diagnosis of diabetes [39, 40]. The underlying construct of diabetes distress overlaps with symptoms of major depression such as fatigue, anhedonia and dysphoria which may further result in functional impairment and associate with poor glycaemic control, poor self-management and increased risk of diabetes related complications [41]. Obesity and unfavorable state of health points to sustained increase in inflammatory activity as a common link between T2D and depression [42]. Here the cognitive function seems to be modulated through metabolic components in regulating fasting glucose. Furthermore, inflammation can trigger behavioral consequences (ex. insomnia, anxiety, and fatigue). We tested the difference in c-reactive protein (CRP), a marker of inflammation, between the three groups and found it was significantly higher in the comorbidity group compared to others in our study (Table 1). Palmos et al. have identified genetic correlation between higher BMI and inflammatory markers, suggesting a common genetic etiology [43]. As such, there is also causal evidence for a bidirectional relationship between BMI and social adversity [44], which may explain the confounding influence of lifestyle factors in our associations. This further corroborates with recent genetic and epigenetic studies that provided conflicting evidence, attributing the cause of comorbidity between T2D and depression largely to shared environmental influences occurring in adulthood [45, 46]. The different environmental factors may activate common pathways and therefore, the comorbidity may be attributed, at least in part, to health behaviors acting on the biological processes.

### Strengths and limitations

We acknowledge our study had strengths as well as some limitations. Notably, this is the first study of its kind to explore the longitudinal influence of prospectively measured adiposity measures on T2D and depression and both as a comorbidity. We unfolded pathways channeled via genetic and lifestyle variability. We included a large dataset from birth with a long follow-up period and data linking to national registers and school records enabling us to adopt a lifecourse approach. One of the major challenges in interpreting diabetes-related studies is the role of nutrition, however, our study population was from a contemporary setting, in which individuals had less obesogenic environmental confounders. To overcome missing data due to longitudinal design, we included complete cases and maximum likelihood estimator in path analysis to impute. The relatively small sample size of T2D and comorbidity may have underpowered our study in identifying certain associations. However, our power calculation for each of the three conditions justifies the sample size and matches the observed effect size in our study (Supplementary Table S8). Heterogeneity in the definition of depression and T2D may have led to misrepresentation of the disease as accuracy of tests vary between populations. Additionally, using different methods of assessment to measure T2D and depression, could have led to differences in capturing the severity of the disease. Our study lacked adiposity measures from the pubertal period which is an important period of habit development and modulating risk in adulthood. We have used a clinical approach based on conventional cut-offs to assess categories of risk factors, however more research is warranted to understand the depth of non-linearity of relationship from a lifecourse perspective using different methods.

## Conclusions

The findings from this study have potential clinical relevance. We did not observe early life shared risk factors between ‘T2D-depression’ comorbidity however, they seem to co-occur due to shared social and lifestyle factors. The underlying mechanism for T2D may work through sequences of biological risks (fetal programming, restricted growth, and weight changes) and the behavioral chain of risk (patterned through lifestyle choices) but depression alone unraveled different origins and pathways in our study. The risk of development of T2D and comorbidity associated with early life adiposity were sensitive to smoking, education, PA, and diet. We triangulated findings using linear relationships of adiposity measures as well as by using overweight categories to understand dichotomy of the relationship. Understanding the underlying mechanism ‘T2D-depression’ comorbidity would be useful in future research to provide further insight on the potential biological mechanism linking them that may be accessible to interventions.

## Supplementary information

Online supplemental material

## Data Availability

NFBC data is available from the University of Oulu, Infrastructure for Population Studies. Permission to use the data can be applied for research purposes via electronic material request portal. In the use of data, we follow the EU general data protection regulation (679/2016) and Finnish Data Protection Act. The use of personal data is based on cohort participant’s written informed consent at his/her latest follow-up study, which may cause limitations to its use. Please, contact NFBC project center (NFBCprojectcenter(at)oulu.fi) and visit the cohort website for more information.
