# Supplementary material for: The relationship of life-course patterns of adiposity with type 2 diabetes, depression, and their comorbidity in the Northern Finland Birth Cohort 1966
Source: Int J Obes (Lond). 2022 May 13;46(8):1470–7. doi: 10.1038/s41366-022-01134-y (PMC9105590; doi:10.1038/s41366-022-01134-y)
Supplement: Supplementary file 1 — Online supplemental material [file 41366_2022_1134_MOESM1_ESM.docx]

**Supplementary Appendix**

**Table of Contents**

[**Supplementary methods** 2](#_Toc98337729)

[**Figure S1. Flowchart of participants included in the study.** 3](#_Toc98337730)

[**Figure S2. Theoretical potential pathways between adiposity variables over the life course with type 2 diabetes, depression, and comorbidity.** 4](#_Toc98337731)

[**Table S1. The test of proportional hazard assumption for each of the covariate in the study.** 5](#_Toc98337732)

[**Table S2. Hazard risk ratio estimates of risk of three outcomes with changes in patterns of** 6](#_Toc98337733)

[**Table S3. Association of categorized birthweight with three main outcomes as well as sequentially adjusted for sex, low education, low PA and unhealthy diet score** 7](#_Toc98337734)

[**Table S4. Hazard risk ratio estimates of three outcomes with patterns of underweight at different time points** 8](#_Toc98337735)

[**Table S5. Distribution of mean of standardized Polygenic Risk Score of BMI between overweight patterns at different time points.** 9](#_Toc98337736)

[**Table S6. Estimates for the total, direct and indirect standardized effects of the pathways of adiposity measures over the life-course on the outcomes** 10](#_Toc98337737)

[**Table S7. Hazard risk ratio of three outcomes with patterns of overweight at 7y, 31y and at both 7y&31y and sequentially adjusted for covariates in all the participants** 11](#_Toc98337738)

[**Table S8. Power calculation of sample size of three conditions** 12](#_Toc98337739)

# **Supplementary methods**

**Testing of assumptions for the Cox regression models**

At first the shape of the associations were tested using linear vs categorical BMI measures. There was a deviation from linearity in some associations, and for clarification both BMI as continuous and categorical variables (BMI categorized as normal and overweight using IOTF criteria) were included in the subsequent analysis. Non-linearity is not an issue for categorical variables which were assessed in the cox regression. Second, the proportional hazard (PH) assumption of the Cox model was tested using statistical tests and diagnostics based on scaled Schoenfeld residuals [function cox.zph() and the output of the test is given below for each covariate. The test for each of the covariates and the global test is statistically non-significant which showed assumption of the proportional hazard to be supported (supplementary table S1).

**Underweight category analysis**

The biological pathways from the underweight category differ from overweight on the health outcomes and differ from the objective of this study. In our main analysis we noted there is no association between BMI measures and overweight with depression. Therefore, for further clarification we tested the associations of the three outcomes with underweight category but did not include this as main part of the analysis. The findings are presented in the supplementary table S4. Overall, it showed that underweight at 7y only and at 31y only was positively associated with depression, but no association was observed when individuals were underweight at both 7y and 31y. Upon adjustments for covariates, the association of underweight at 7y with depression disappeared.

# **Figure S1. Flowchart of participants included in the study.**


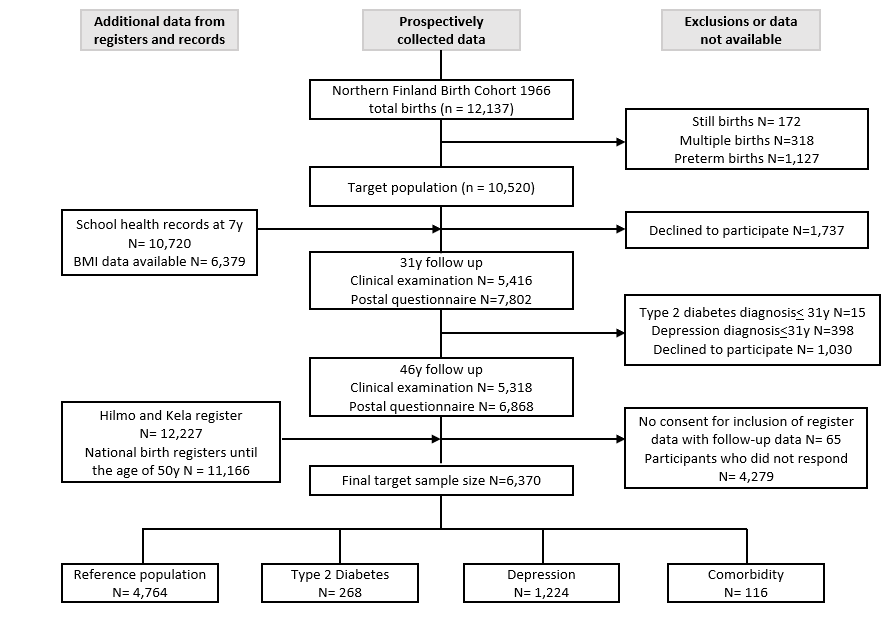


# **Figure S2. Theoretical potential pathways between adiposity variables over the life course with type 2 diabetes, depression, and comorbidity.**


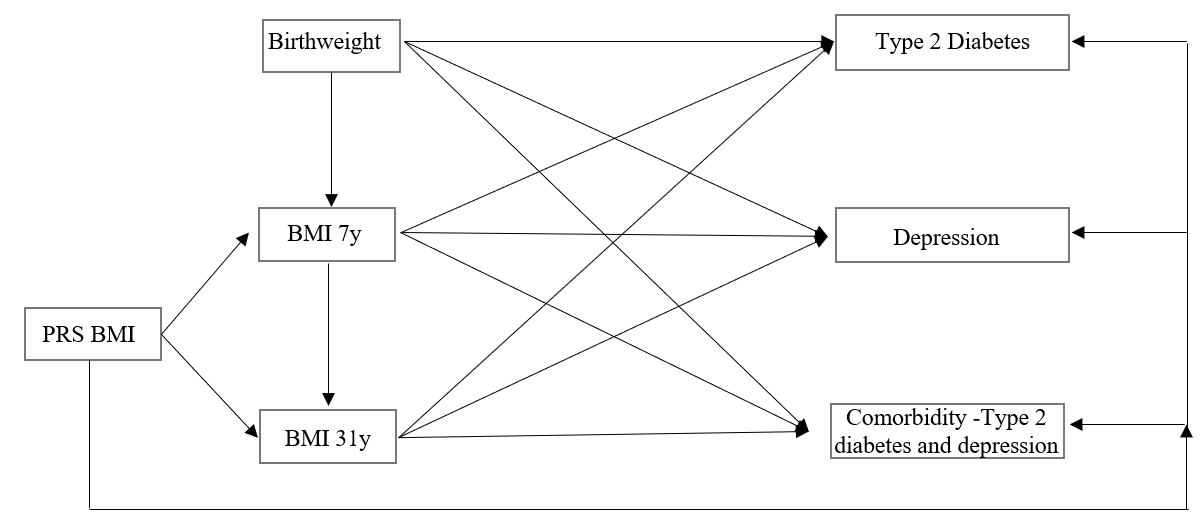


# **Table S1. The test of proportional hazard assumption for each of the covariate in the study.**

| **Variable** | **Chi-sq** | **P-value** |
| --- | --- | --- |
| Overweight pattern | 4.49 | 0.21 |
| Sex | 6.72 | 0.07 |
| Education | 0.40 | 0.52 |
| Smoking 31y | 0.06 | 0.81 |
| Physical activity | 0.34 | 0.56 |
| Diet | 3.44 | 0.06 |
| Global test | 9.81 | 0.20 |

**Table S2. Hazard risk ratio estimates of risk of three outcomes with changes in patterns of overweight at different time points and stratification by sex.**

|  | **All participants** | | | | **Females** | | | | **Males** | | | |
| --- | --- | --- | --- | --- | --- | --- | --- | --- | --- | --- | --- | --- |
|  | **Model 1** |  | **Model 2** |  | **Model 1** |  | **Model 2** |  | **Model 1** |  | **Model 2** |  |
| **Type 2 Diabetes** | **HR (95% CI)** | **P-value** | **HR (95% CI)** | **P-value** | **HR (95% CI)** | **P-value** | **HR (95% CI)** | **P-value** | **HR (95% CI)** | **P-value** | **HR (95% CI)** | **P-value** |
| No overweight | Reference | - | Reference | - | Reference | - | Reference | - | Reference | - | Reference | - |
| Overweight 7y | 2.1 (1.2, 3.7) | 0.008 | 1.3 (0.5, 3.1) | 0.61 | 2.3 (0.9, 5.3) | 0.06 | 1.1 (0.3, 4.7) | 0.87 | 2.1 (1.0, 4.3) | 0.05 | 1.5 (0.5, 4.7) | 0.52 |
| Overweight 31y | 3.1 (2.4, 4.0) | <0.001* | 3.1 (2.3, 4.1) | <0.001* | 4.7 (3.1, 7.0) | <0.001* | 4.3 (2.7, 7.0) | <0.001* | 2.1 (1.5, 2.9) | <0.001 | 2.4 (1.6, 3.5) | <0.001 |
| Overweight at both ages | 3.8 (2.4, 6.1) | <0.001* | 3.1 (1.7, 5.4) | <0.001* | 5.3 (2.8, 10.3) | <0.001* | 4.8 (2.2, 10.6) | <0.001* | 2.8 (1.5, 5.5) | 0.002 | 2.0 (0.9, 4.7) | 0.11 |
| **Depression** | | | | | | | | | | | | |
| No overweight | Reference | - | Reference | - | Reference | - | Reference | - | Reference | - | Reference | - |
| Overweight 7y | 1.2 (0.9, 1.5) | 0.11 | 1.3 (0.9, 1.8) | 0.16 | 1.2 (0.9, 1.6) | 0.31 | 1.4 (0.9, 2.0) | 0.13 | 1.1 (0.63, 1.8) | 0.85 | 1.1 (0.6, 2.2) | 0.75 |
| Overweight 31y | 1.0 (0.9, 1.2) | 0.76 | 1.1 (0.9, 1.3) | 0.20 | 1.2 (0.9, 1.4) | 0.09 | 1.2 (0.9, 1.4) | 0.15 | 1.0 (0.8, 1.3) | 0.78 | 1.0 (0.8, 1.3) | 0.72 |
| Overweight at both ages | 1.1 (0.8, 1.5) | 0.15 | 1.2 (0.9, 1.7) | 0.26 | 1.0 (0.7, 1.5) | 0.88 | 1.2 (0.8, 1.8) | 0.52 | 1.2 (0.7, 2.0) | 0.43 | 1.3 (0.8, 2.2) | 0.34 |
| **Comorbidity - Type 2 Diabetes with depression** | | | | | | | | | | | | |
| No overweight | Reference | - | Reference | - | Reference | - | Reference | - | Reference | - | Reference | - |
| Overweight 7y | 2.8 (1.5, 5.5) | 0.002 | 1.3 (0.3, 5.5) | 0.71 | 1.5 (0.4, 6.5) | 0.56 | 1.3 (0.3, 5.5) | 0.71 | 2.8 (0.8, 9.5) | 0.09 | 3.1 (0.7, 13.5) | 0.14 |
| Overweight 31y | 3.8 (2.7, 5.4) | <0.001* | 3.6 (2.2, 5.7) | <0.001* | 4.9 (2.3, 8.7) | <0.001* | 3.2 (1.9, 5.1) | <0.001* | 2.6 (1.4, 4.8) | 0.002 | 2.4 (1.2, 4.8) | 0.01 |
| Overweight at both ages | 5.0 (2.7, 9.5) | <0.001* | 3.2 (1.3, 7.6) | <0.01* | 5.8 (2.4, 14.3) | <0.001* | 3.4 (1.4, 8.1) | <0.001* | 4.1 (1.4, 12.1) | 0.01 | 2.1 (0.5, 9.0) | 0.33 |

Model 1: Unadjusted; Model 2: Adjusted for low education, smoking, low physical activity and unhealthy diet score at 31y and additionally for sex in the ‘all participants’ group. * signifies *P*-value<0.05.CI: Confidence Interval and HR: Hazard Ratio

**Table S3. Association of categorized birthweight with three main outcomes as well as sequentially adjusted for sex, low education, low PA and unhealthy diet score.**

|  | Type 2 Diabetes | | Depression | | Comorbidity | |
| --- | --- | --- | --- | --- | --- | --- |
|  | OR (95% CI) | P-value | OR (95% CI) | P-value | OR (95% CI) | P-value |
| Birthweight 1 | 1.19 (0.81, 1.75) | 0.28 | 1.10 (0.79, 1.54) | 0.84 | 2.51 (1.24, 5.07) | 0.02* |
| Birthweight 2 | 1.02 (0.82, 1.23) | 0.75 | 1.20 (1.02, 1.41) | 0.56 | 1.04 (0.63, 1.73) | 0.69 |
| Birthweight 4 | 0.88 (0.69, 1.10) | 0.39 | 1.12 (0.95, 1.32) | 0.85 | 0.68 (0.38, 1.19) | 0.06 |
| Birthweight 5 | 0.71 (0.46, 1.09) | 0.11 | 1.02 (0.77, 1.34) | 0.39 | 0.95 (0.39, 2.24) | 0.60 |
| Birthweight 6 | 1.16 (0.42, 3.21) | 0.70 | 1.44 (0.70, 2.95) | 0.44 | 1.45 (0.19, 10.79) | 0.79 |
| Adjusted for sex | | | |  |  |  |
| Birthweight 1 | 1.64 (1.04, 2.57) | 0.02* | 1.07 (0.76, 1.50) | 0.57 | 2.52 (1.25, 5.11) | 0.02* |
| Birthweight 2 | 1.08 (0.83, 1.41) | 0.47 | 1.15 (0.98, 1.36) | 0.89 | 1.05 (0.63, 1.75) | 0.73 |
| Birthweight 4 | 0.82 (0.62, 1.08) | 0.32 | 1.17 (0.99, 1.38) | 0.96 | 0.67 (0.38, 1.18) | 0.05 |
| Birthweight 5 | 0.77 (0.48, 1.24) | 0.33 | 1.12 (0.85, 1.48) | 0.75 | 0.93 (0.39, 2.21) | 0.58 |
| Birthweight 6 | 0.71 (0.17, 2.97) | 0.62 | 1.56 (0.76, 3.22) | 0.35 | 1.42 (0.19, 10.62) | 0.80 |
| Adjusted for low education | | | |  |  |  |
| Birthweight 1 | 1.00 (0.59, 1.70) | 0.70 | 1.18 (0.81, 1.72) | 0.64 | 1.57 (0.61, 4.08) | 0.35 |
| Birthweight 2 | 1.07 (0.82, 1.39) | 0.32 | 1.12 (0.92, 1.35) | 0.81 | 0.99 (0.56, 1.77) | 0.84 |
| Birthweight 4 | 0.83 (0.63, 1.10) | 0.59 | 1.12 (0.93, 1.35) | 0.77 | 0.66 (0.35, 1.27) | 0.14 |
| Birthweight 5 | 0.74 (0.45, 1.21) | 0.39 | 0.93 (0.67, 1.29) | 0.31 | 0.79 (0.28. 2.23) | 0.53 |
| Birthweight 6 | 0.86 (0.20, 3.59) | 0.92 | 1.20 (0.52, 2.79) | 0.78 | 1.68 (0.22, 12.67) | 0.58 |
| Adjusted for low physical activity | | | | | | |
| Birthweight 1 | 1.06 (0.53, 2.13) | 0.69 | 1.15 (0.80, 1.64) | 0.84 | 2.38 (1.09, 5.20) | 0.03* |
| Birthweight 2 | 1.08 (0.76, 1.52) | 0.49 | 1.19 (1.00, 1.43) | 0.42 | 1.00 (0.58, 1.75) | 0.79 |
| Birthweight 4 | 0.93 (0.66, 1.33) | 0.97 | 1.08 (0.90, 1.29) | 0.78 | 0.55 (0.29, 1.08) | 0.04* |
| Birthweight 5 | 0.92 (0.51, 1.67) | 0.99 | 0.88 (0.64, 1.19) | 0.10 | 0.72 (0.25, 2.03) | 0.37 |
| Birthweight 6 | 0.64 (0.09, 4.73) | 0.67 | 1.43 (0.67, 3.05) | 0.43 | 1.67 (0.22, 12.6) | 0.61 |
| Adjusted for unhealthy diet score | | | | | | |
| Birthweight 1 | 1.07 (0.53, 2.16) | 0.69 | 1.18 (0.83, 1.69) | 0.67 | 2.45 (1.12, 5.36) | 0.03* |
| Birthweight 2 | 1.09 (0.77, 1.54) | 0.49 | 1.22 (1.03, 1.46) | 0.29 | 0.98 (0.56, 1.72) | 0.59 |
| Birthweight 4 | 0.92 (0.65, 1.31) | 0.94 | 1.09 (0.91, 1.30) | 0.87 | 0.57 (0.29, 1.09) | 0.03* |
| Birthweight 5 | 0.67 (0.09, 4.95) | 0.99 | 0.88 (0.65, 1.20) | 0.12 | 0.92 (0.36, 2.37) | 0.60 |
| Birthweight 6 | 0.67 (0.09, 4.95) | 0.69 | 1.31 (0.59, 2.89) | 0.62 | 1.79 (0.24, 13.41) | 0.60 |

Birthweight categories 1 = 2.0–2.7; 2 = 2.7–3.2; 3 = 3.2–3.7; 4 =3.7–4.2; 5 = 4.2–4.7; 6 = 4.7–5.5 kg. Reference category: 3 = 3.2–3.7. * signifies *P*-value<0.05

# **Table S4. Hazard risk ratio estimates of three outcomes with patterns of underweight at different time points**

|  | **Model 1** | | **Model 2** | |
| --- | --- | --- | --- | --- |
| **Type 2 Diabetes** | **HR (95% CI)** | **P-value** | **HR (95% CI)** | **P-value** |
| Underweight at 7y | 1.4 (1.0, 1.8) | 0.04* | 1.4 (0.9, 1.9) | 0.06 |
| Underweight at 31y only | 1.1 (0.8, 1.6) | 0.52 | 0.9 (0.5, 1.4) | 0.59 |
| Underweight at both ages | 0.82 (0.6, 1.2) | 0.30 | 0.8 (0.5, 1.3) | 0.35 |
| **Depression** | | | | |
| Underweight at 7y | 1.2 (1.1, 1.4) | 0.008* | 1.1 (0.96, 1.30) | 0.06 |
| Underweight at 31y only | 1.3 (1.14, 1.6) | 0.001* | 1.3 (1.04, 1.6) | 0.02* |
| Underweight at both ages | 1.1 (0.94, 1.3) | 0.21 | 1.1 (0.90, 1.3) | 0.39 |
| **Comorbidity** | | | | |
| Underweight at 7y | 1.3 (0.78, 2.0) | 0.35 | 1.1 (0.6, 1.9) | 0.72 |
| Underweight at 31y only | 0.9 (0.5, 1.6) | 0.71 | 0.9 (0.4, 1.9) | 0.70 |
| Underweight at both ages | 0.8 (0.5, 1.5) | 0.55 | 0.9 (0.4, 1.8) | 0.73 |

* signifies *P*-value<0.05

# **Table S5. Distribution of mean of standardized Polygenic Risk Score of BMI between overweight patterns at different time points.**

|  | **Mean** | **P-value** |
| --- | --- | --- |
| Overweight at 7y | 0.11 | <0.0001* |
| Overweight at 31y | 0.32 |  |
| Overweight at both ages | 0.60 |  |

* signifies *P*-value<0.05

**Table S6. Estimates for the total, direct and indirect standardized effects of the pathways of adiposity measures over the life-course on the outcomes**.

|  | **Total effect** | **Direct effect** | **Indirect effect** |
| --- | --- | --- | --- |
| **Birthweight** | | | |
| Type 2 diabetes | -0.02* | -0.03* | 0.01* |
| Comorbidity-Type 2 diabetes and depression | -0.03* | -0.05* | 0.02* |
| **BMI 7y** | | | |
| Type 2 diabetes | 0.03* | -0.13* | 0.15* |
| Comorbidity-Type 2 diabetes and depression | 0.07* | -0.04* | 0.12* |
| **BMI 31y** | | | |
| Type 2 diabetes | 0.37* | 0.37* | 0 |
| Comorbidity-Type 2 diabetes and depression | 0.28* | 0.28* | 0 |
| **PRS BMI** | | | |
| Type 2 diabetes | 0.08* | 0 | 0.08* |
| Comorbidity-Type 2 diabetes and depression | 0.03* | 0 | 0.03* |

* signifies *P*-value<0.0001

# **Table S7. Hazard risk ratio of three outcomes with patterns of overweight at 7y, 31y and at both 7y&31y and sequentially adjusted for covariates in all the participants**

|  | **Type 2 diabetes** | | **Depression** | | **Comorbidity - Type 2 diabetes with depression** | |
| --- | --- | --- | --- | --- | --- | --- |
|  | **HR (95% CI)** | **P-value** | **HR (95% CI)** | **P-value** | **HR (95% CI)** | **P-value** |
| Overweight at 7y | 2.1 (1.2, 3.7) | 0.008* | 1.2 (0.9, 1.5) | 0.11 | 2.8 (1.5, 5.5) | 0.002* |
| + sex | 2.1 (1.2, 3.7) | 0.007* | 1.1 (0.9, 1.5) | 0.35 | 2.1 (0.8, 5.3) | 0.11 |
| + low education | 1.2 (0.5, 3.0) | 0.66 | 1.3 (0.9, 1.8) | 0.15 | 1.3 (0.3, 5.5) | 0.71 |
| + smoking | 1.4 (0.7, 3.0) | 0.38 | 1.2 (0.9, 1.7) | 0.21 | 1.2 (0.3, 5.0) | 0.81 |
| + unhealthy diet score | 1.5 (0.7, 3.3) | 0.29 | 1.2 (0.9, 1.7) | 0.19 | 1.3 (0.3, 5.6) | 0.69 |
| + low physical activity | 1.6 (0.7, 3.3) | 0.27 | 1.3 (0.9, 1.8) | 0.09 | 1.3 (0.3, 5.5) | 0.71 |
| Overweight at 31y | 3.1 (2.4, 4.0) | <0.001* | 1.0 (0.9, 1.2) | 0.76 | 3.8 (2.7, 5.4) | <0.001* |
| + sex | 2.9 (2.2, 3.8) | <0.001* | 1.1 (0.9, 1.3) | 0.15 | 3.6 (2.4, 5.5) | <0.001* |
| + low education | 3.3 (2.5, 4.4) | <0.001* | 1.0 (0.9, 1.2) | 0.86 | 3.5 (2.2, 5.6) | <0.001* |
| + smoking | 3.3 (2.5, 4.3) | <0.001* | 1.0 (0.9, 1.2) | 0.56 | 4.0 (2.5, 6.3) | <0.001* |
| + unhealthy diet score | 3.2 (2.4, 4.2) | <0.001* | 1.0 (0.9, 1.2) | 0.65 | 3.9 (2.5, 6.2) | <0.001* |
| + low physical activity | 3.2 (2.5, 4.3) | <0.001* | 1.0 (0.9, 1.2) | 0.56 | 3.8 (2.4, 6.0) | <0.001* |
| Overweight at both ages | 3.8 (2.4, 6.1) | <0.001* | 1.1 (0.8, 1.5) | 0.15 | 5.0 (2.7, 9.5) | <0.001* |
| + sex | 3.8 (2.4, 6.0) | <0.001* | 1.1 (0.8, 1.5) | 0.55 | 5.0 (2.5, 10.0) | <0.001* |
| + low education | 3.4 (2.0, 5.9) | <0.001* | 1.1 (0.8, 1.4) | 0.42 | 4.8 (2.2, 10.4) | <0.001* |
| + smoking | 3.8 (2.3, 6.1) | <0.001* | 1.1 (0.8, 1.5) | 0.47 | 4.9 (2.3, 10.3) | <0.001* |
| + unhealthy diet score | 3.8 (2.3, 6.1) | <0.001* | 1.1 (0.8, 1.5) | 0.45 | 5.3 (2.5, 11.2) | <0.001* |
| + low physical activity | 4.0 (2.5, 6.5) | <0.001* | 1.2 (0.8, 1.6) | 0.37 | 4.5 (2.1, 9.9) | <0.001* |

In the calculation of hazard ratio (HR) for the risk of outcomes, participants who had no overweight were used as reference group. CI denotes confidence interval. * signifies *P*-value<0.05

# **Table S8. Power calculation of sample size of three conditions**

| Exposure | N cases | N control | Effect size | Significance level | Test | Power |
| --- | --- | --- | --- | --- | --- | --- |
| Type 2 diabetes | 268 | 4764 | 0.18 | 0.05 | Two-sided | 0.8 |
| Depression | 1224 | 4764 | 0.09 | 0.05 | Two-sided | 0.8 |
| Comorbidity | 116 | 4764 | 0.26 | 0.05 | Two-sided | 0.8 |
